# Trends in Hospitalizations of Patients with Idiopathic Inflammatory Myopathies: Long-Term Observational Study, 2010–2024

**DOI:** 10.3390/jcm15197707

**Published:** 2026-10-05

**Authors:** Maria Dutsch-Wicherek, Bartłomiej Wieleba, Katarzyna Lewtak, Krzysztof Kanecki, Jakub Stokwiszewski, Małgorzata Wisłowska

**Affiliations:** 1 Faculty of Medicine, Medical University of Warsaw, 02-091 Warsaw, Poland; 2 Department of Social Medicine and Public Health, Medical University of Warsaw, 02-091 Warsaw, Poland; 3 National Institute of Public Health NIH—National Research Institute, 00-791 Warsaw, Poland; 4 National Institute of Geriatrics, Rheumatology and Rehabilitation, 02-637 Warsaw, Poland

**Keywords:** dermatomyositis, idiopathic inflammatory myopathy, juvenile dermatomyositis, mortality, polymyositis

## Abstract

**Background/Objectives:** Idiopathic inflammatory myopathies (IIMs) are very rare, chronic, immune-mediated diseases that clinically manifest with myositis symptoms and extramuscular manifestations. The rarity of the disease makes it difficult to conduct double-blind studies, and large population-based studies are needed to improve our knowledge of them. This study aims to analyze the hospitalization rates and trends of IIMs between 2010 and 2024. Additionally, a characterization of the IIM patient population in Poland was conducted. **Methods**: A retrospective study using data from the Nationwide General Hospital Morbidity Study (NGHMS) between 2010 and 2024 was conducted. Data included demographic characteristics, principal diagnosis, hospitalization, and in-hospital mortality. Classification in accordance with ICD-10 was applied. A total of 7139 patients were included. Appropriate descriptive analyses, comparative tests, Poisson regression for incidence trends, and logistic regression for mortality predictors were performed, with *p* < 0.05 considered statistically significant. **Results:** The median IIM incidence in the Polish population was 1.209 per 100,000 per year. The overall female-to-male ratio was 1.68. Hospitalization and mortality trends for IIM were stable over the time period. The incidence among voivodeships in Poland was comparable. **Conclusions**: Nationwide, comprehensive data are an extremely valuable tool for researching and understanding diseases as rare as IIMs. The incidence, hospitalization, and mortality rates of IIMs in Poland were stable between 2010 and 2024.

## 1. Introduction

Idiopathic inflammatory myopathies (IIMs) are a diverse group of chronic, immune-mediated muscle diseases. Myopathy symptoms include progressive muscle weakness, myalgia, and fatigue. While muscle symptoms predominate, extramuscular involvement such as cutaneous, articular, pulmonary, cardiac, and gastrointestinal manifestations is frequent and can significantly affect morbidity and prognosis. Organ involvement strongly influences outcomes [1]. Disease incidence varies widely, reflecting both true epidemiological differences and diagnostic challenges [1,2].

The current, most widely used IIM classification is based on the 2017 EULAR criteria [3], which distinguish several subtypes: juvenile dermatomyositis (JDM), dermatomyositis (DM), clinically amyopathic dermatomyositis (CADM), inclusion body myositis (IBM), polymyositis (PM), and immune-mediated necrotizing myopathy (IMNM). Proper diagnosis is crucial for management, though some subtypes remain difficult to distinguish and misdiagnosis persists. Newer, more detailed classifications have also been proposed, including antisynthetase syndrome (ASyS) and overlap myositis (OM) with other connective tissue diseases, as developed by Gianni et al. [4].

Women are more commonly affected by DM, PM, and IMNM, whereas IBM is more prevalent in men. An IIM diagnosis includes clinical, serological, and pathological data, with myositis-specific autoantibodies (MSAs)—including anti-Jo-1, anti-Mi-2, anti-MDA5, anti-TIF1-gamma, and anti-NXP2—correlating with certain clinical features. Anti-Jo-1 is the most common antibody. Anti-Mi-2, anti-NXP2, and anti-TIF-1 correlate with malignancy. Anti-Jo-1 and anti-MDA5 antibodies are associated with the risk of interstitial lung disease (ILD) [5].

Laboratory test results include markers of myopathy, including elevated creatine kinase (CK) and aldolase levels, though normal levels do not exclude the diagnosis [1,2]. CK is more sensitive and can be used to monitor disease activity. Electromyography and muscle magnetic resonance imaging (MRI) support diagnosis, while muscle biopsy remains an important diagnostic tool for IIM, especially in ambiguous or seronegative cases. Biopsy must be performed under ultrasound or MRI guidance to increase precision [1,6]. Lung involvement, especially ILD, as well as pulmonary hypertension, is a frequent and serious extramuscular manifestation of IIM, most notably in DM, CADM, ASyS and PM, and it is associated with a poorer prognosis and increased mortality [7].

The management of IIM requires further research, as the rarity of the disease makes it difficult to conduct double-blind studies. Immunosuppressive therapy should be tailored according to disease severity and organ involvement. Glucocorticosteroids form the cornerstone of treatment and are frequently used in combination with steroid-sparing agents, such as methotrexate, azathioprine, mycophenolate mofetil, or rituximab. Newer targeted therapies are emerging, yet further data are needed to establish guidelines for their use [2].

In the present study, data from the Nationwide General Hospital Morbidity Study (NGHMS) were retrospectively analyzed. The database implements the ICD-10 classification, as used by the Polish NFZ (Narodowy Fundusz Zdrowia; in English, National Health Fund), which recognizes only DM, PM, JDM, and “unspecified dermatopolymyositis”, an umbrella term for patients who do not meet the criteria for the three aforementioned conditions. According to the Bohan and Peter classification [8], IMNM, OM, and ASyS were all classified as PM, and in our study, these subtypes likely appear as PM.

As such, we include a brief characterization of DM, PM, and JDM.

With this study, we aim to characterize the demographic structure of IIM patients in Poland, identify possible geographical regions with higher IIM incidence, and describe hospitalization and incidence rates.

### 1.1. Dermatomyositis

Dermatomyositis is characterized by progressive, symmetric muscle weakness and distinctive cutaneous rashes, including Gottron’s papules and a heliotrope rash. Chronic inflammation results in muscle fibrosis and atrophy, and delayed diagnosis may cause irreversible damage and disability. Extramuscular manifestations, such as ILD and cardiovascular manifestations, are also frequently observed [9]. In DM, CD4+ T cells and B lymphocytes play a key role. They damage blood vessels and muscle fibers and primarily accumulate around blood vessels, forming perivascular infiltrates [10].

The following autoantibodies are observed in DM: anti-Mi-2, anti-TIF-1γ, anti-MDA-5, and anti-NXP2. The presence of anti-Mi-2 autoantibodies is associated with a favorable prognosis [9]. DM with anti-MDA-5 autoantibodies is associated with cutaneous ulcers, severe non-scarring alopecia, painful erythematous macules on the palms or palmar surfaces of the interphalangeal joints, mechanic’s hands, arthritis, and ILD [8].

Calcinosis cutis correlates with disease severity. Calcium deposits occur on the limbs and trunk, forming in areas previously affected by the disease or within the subcutaneous tissue. Complications include infection, ulceration, and restricted joint mobility, which correlate with the presence of anti-NXP2 autoantibodies [8,9].

Dermatomyositis is associated with an increased risk of malignancies, most frequently ovarian, lung, colorectal, and pancreatic cancers, as well as non-Hodgkin lymphoma. This association is most commonly linked to the presence of anti-TIF-1γ and anti-NXP2 antibodies. The anti-TIF-1γ antibody is most common in patients with DM and a risk of cancer, so it is important to perform screening tests within the first 3 years after symptoms appear [1,11]. In some cases, successful oncological treatment may improve DM symptoms, although further research is needed.

Established risk factors for developing DM include ultraviolet (UV) radiation exposure, the HLA-8.1 allele, and the non-HLA alleles PLCL1 and BLK [1].

### 1.2. Polymyositis

Polymyositis is a rare subtype of IIM, accounting for less than 2% of cases, and it is characterized by muscle involvement without cutaneous manifestations. PM presents with symmetric proximal muscle weakness and is often regarded as a heterogeneous and poorly defined clinical entity [12].

CD8+ cytotoxic T lymphocytes infiltrate muscle tissue, damaging it. Histopathological images are diverse and crucial, primarily for differentiating PM from IBM, which is also characterized by such invasion [10,12]. The most significant finding is the absence of rimmed vacuoles and amyloid deposits. Other differential diagnoses include IMNM, OM, and ASyS. It is very important to note that, according to the Bohan and Peter classification [8], these conditions are all classified as PM, and in our work, these subtypes likely appear as PM. There are no MSA or MAA (myositis-associated antibodies, including anti-PM-Scl, anti-Ku, anti-U1RNP, and anti-Ro52) antibodies specific to PM, yet detecting such autoantibodies has enabled proper verification of the diagnosis in patients previously diagnosed with PM. Established risk factors for developing PM include hepatitis B infection, the HLA-8.1 allele, and the non-HLA allele PTPN22. Human immunodeficiency virus (HIV) and human T-lymphotropic virus type 1 (HTLV-1) infections can also trigger PM [1].

### 1.3. Juvenile Dermatomyositis

Juvenile dermatomyositis (JDM) is a very rare IIM with an incidence of 2–4 per million, and it is the most common IIM in pediatric patients. Early detection, diagnosis, and identification of the clinical phenotype are crucial for proper treatment and establishing a prognosis [13,14].

Several classification systems exist for JDM. Traditionally, monocyclic disease (symptoms subside within two years of diagnosis), chronic persistent disease (active beyond two years), and polycyclic disease (at least one flare-up after six months of remission) have been distinguished. However, this classification requires modification, as polycyclic patients have been shown to exhibit subclinical epithelial injury [14].

The new classification approach focuses on the clinical phenotype and its correspondence to specific autoantibodies, which is consistent with the current division of IIM. The dermatomyositis phenotype can be represented by a few autoantibodies, such as anti-Mi-2, anti-NXP2, anti-TIF1, anti-MDA5, and anti-Ro52 [15]. Anti-Mi-2 correlates with less severe and shorter disease courses and lower mortality. Muscle involvement is severe and confirmed by pathology findings. This phenotype responds well to standard treatment and is likely to enter remission [13,14,15]. Anti-NXP2 and anti-TIF1 correlate with a more severe disease course, and their presence corresponds with lower remission rates. The anti-NXP2 phenotype is characterized by marked muscle and gastrointestinal involvement, as well as calcinosis. Anti-TIF1 correlates with milder muscle involvement, yet more severe skin symptoms.

In contrast to adult DM patients, this phenotype in the pediatric population has not been shown to correlate with malignancy [14,15]. Anti-MDA5 positivity is associated with persistent disease requiring continuous therapy, even in the presence of less severe muscle involvement. This phenotype, especially when coexisting with anti-Ro52, carries a greater risk of developing ILD [13].

Current treatment recommendations, as outlined by Bellutti Enders et al., emphasize the lack of head-to-head or superiority trials for most therapeutic regimens, primarily due to small patient populations and variable clinical phenotypes. Initial therapy with high-dose corticosteroids combined with disease-modifying agents such as methotrexate or ciclosporin A (CsA) has demonstrated the most favorable outcomes. Refractory disease has been successfully managed with intravenous immunoglobulin (IVIG), cyclophosphamide, CsA, azathioprine, mycophenolate mofetil, hydroxychloroquine, and tacrolimus [16].

## 2. Patients and Methods

Data were obtained from the Nationwide General Hospital Morbidity Study (NGHMS), the principal national source of individual-level hospital morbidity data in Poland, conducted by the National Institute of Public Health NIH—National Research Institute (NIPH NIH-NRI) within the Program of Statistical Surveys of Official Statistics. The NGHMS collects data from both public and private hospitals (excluding psychiatric wards) and covers all patients admitted to the hospital, regardless of their place of residence or insurance status.

The data were collected from 1 January 2010 to 31 December 2024.

The collected data enable a comprehensive analysis of the structure and frequency of hospitalizations. The following data were collected for each patient:

- Patient characteristics, such as sex, age, and place of residence.
- Principal diagnosis (the reason behind the patient’s hospitalization) according to the International Statistical Classification of Diseases and Related Health Problems, 10th Revision (ICD-10):
  ○ M33.0 for juvenile dermatomyositis;
  ○ M33.1 for other dermatomyositis (i.e., DM);
  ○ M33.2 for polymyositis;
  ○ M33.9 for dermatopolymyositis, unspecified.
- Information on hospitalization: admission and discharge dates, including in-hospital mortality.

Patients included in the study were those diagnosed with DM, PM, JDM, and “unspecified myopathies”. All the aforementioned data were obtainable. A total of 113 patients were disqualified from the study due to probable miscoding of diagnoses in the database (JDM diagnoses assigned to patients aged over 18 years in non-rheumatological wards). No further patients were disqualified due to unmet criteria, data inconsistencies, or unavailability.

The analyzed data are presented as median (IQR) and categorical data as n (%). For comparisons, the χ^2^ test or Fisher’s exact test was used; the percentage of women was evaluated using the binomial test vs. 50%; and age and length of hospitalization were compared using the Mann–Whitney U test. Regional (voivodeship) variation was assessed using the χ^2^ goodness-of-fit test with a Benjamini–Hochberg correction, and incidence trends were assessed using Poisson regression. Predictors of death (age and sex) were analyzed using logistic regression. A *p*-value < 0.05 was considered statistically significant.

The study protocol adhered to the principles of the Declaration of Helsinki and Good Clinical Practice. Approval was granted by the institutional bioethics committee (KBT-4/1/2026, 28 May 2026). This study did not involve direct human participation. No informed consent was obtained because the research conducted was a secondary analysis of a large, pre-existing national healthcare dataset. All data extracted from the NGHMS database were anonymized and did not contain any information that could allow patient identification. The research team had no access to any identifiable patient information. Therefore, there were no means of identifying individual patients or linking the analyzed records to identifiable persons. No patient was contacted by the investigators, and no additional information was obtained from patients for this study.

## 3. Results

The analysis identified 7139 patients diagnosed with DM, PM, JDM, and “unspecified myopathies” in the database. The sex distribution in the entire cohort is presented in Table 1.

Sex and age distribution among the subcohorts are presented in Table 2. Females were predominantly affected by all IIM subtypes. The overall female-to-male ratio was 1.68.

PM was by far the most common diagnosis (3220 patients), followed by unspecified myopathies (1919 patients) and DM (1730 patients). JDM was the rarest diagnosis, with only 270 patients. The median age of patients with JDM (11; range 6–14) was naturally markedly lower than that of the others. The median age of patients with other diagnoses was similar: 56 (range 41–66) for DM and unspecified myopathies and 57 (range 41–67) for PM.

Duration of stay and in-hospital mortality characteristics for each IIM subtype are presented in Table 3. The median duration of stay for all patients was 7 days. A total of 118 patients died during admission due to the primary diagnosis. The specific subtype was not associated with mortality (χ^2^ *p* = 0.14). No child (<18 years) died. Patients who died were markedly older than survivors (median 68 vs. 55 years; Mann–Whitney *p* = 5.6 × 10^−19^) and were hospitalized for a longer time (median 18 vs. 7 d).

**Figure 1:**
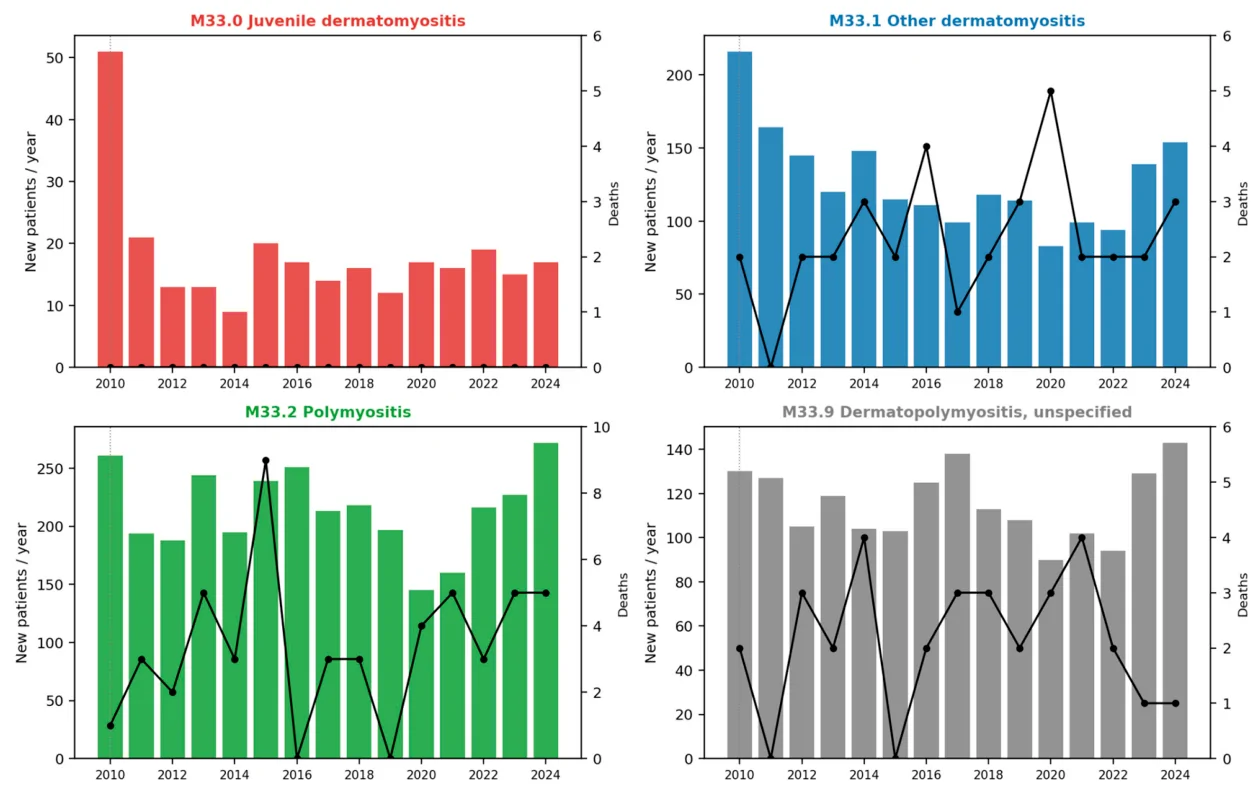
Annual first hospitalizations and in-hospital deaths by M33 subtype. One panel per diagnosis. Bars = new hospitalizations/year (**left** axis); black line = in-hospital deaths (**right** axis). No deaths occurred in M33.0 (all patients < 18 years).

Figure 1 shows the trend lines for the number of admissions and deaths per year by diagnosis. Hospitalization and mortality rates did not meaningfully correlate. A small dip in hospitalization rates at the peak of the COVID-19 pandemic was noted in all IIM subtypes. Aside from that, hospitalization rates for all subtypes remained stable over the period. Mortality rates rose for DM, unspecified myopathies, and PM in the year 2020, possibly due to the COVID-19 pandemic.

**Figure 2:**
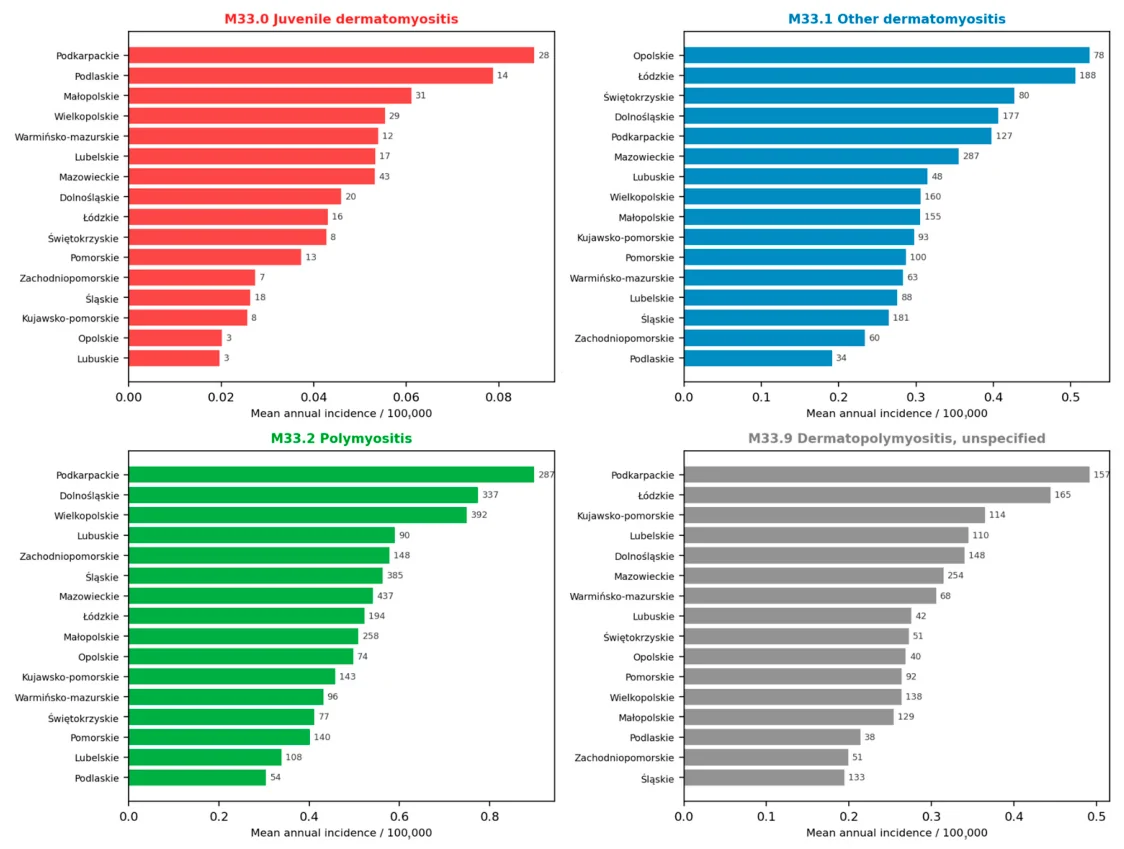
Mean annual incidence of M33 per 100,000 (bars) by voivodeship of residence and subtype; numbers at bar ends = absolute patient counts.

Figure 2 presents the mean annual incidence of specific IIMs by place of residence (voivodeship). Podkarpackie Voivodeship had the highest mean annual incidence of JDM, PM, and unspecified myopathies (around 0.85, 0.85, and 0.5 per 100,000, respectively). The highest mean annual incidence of DM was observed in Opolskie Voivodeship (around 0.5 per 100,000).

**Figure 3:**
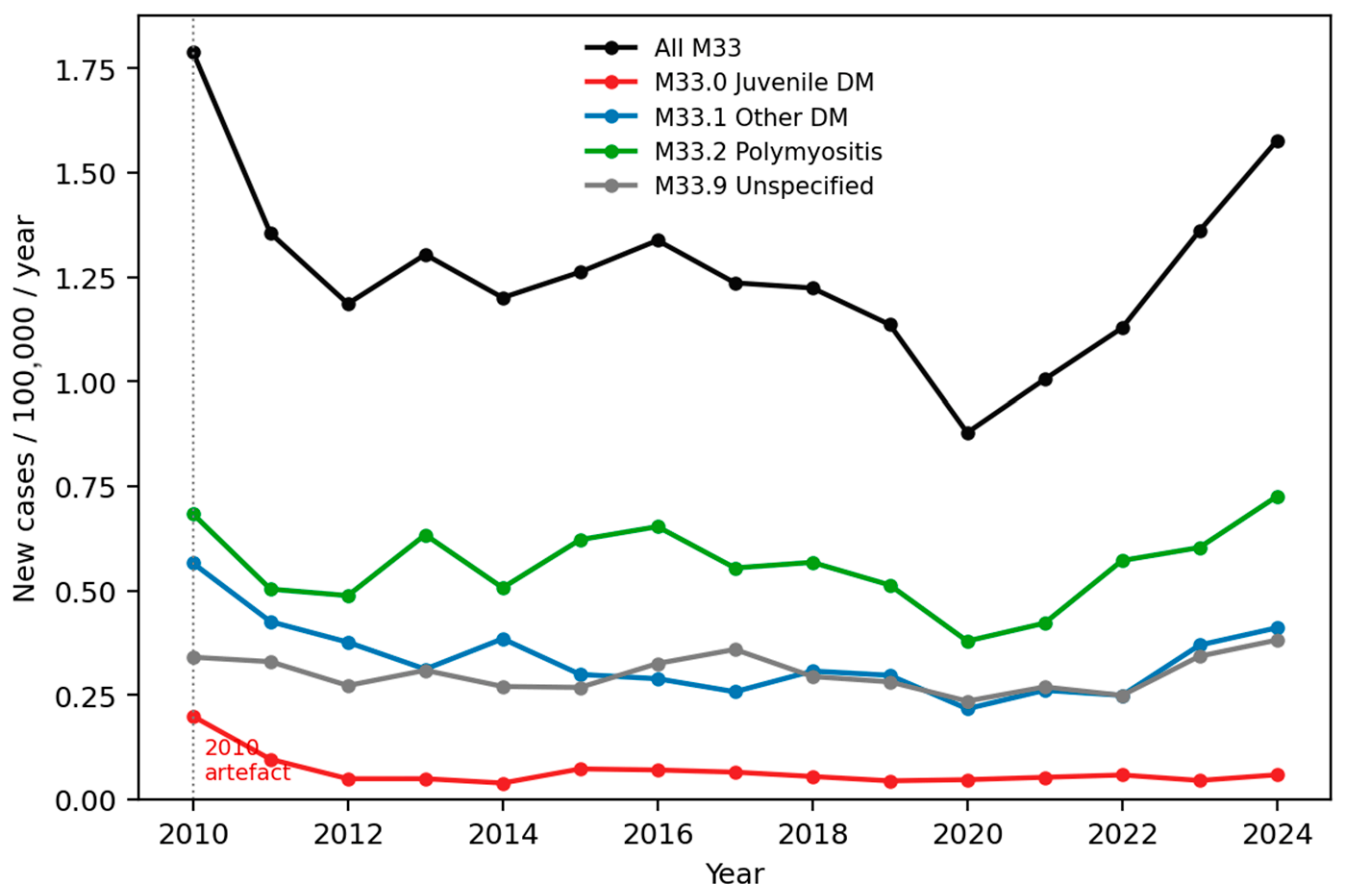
Annual incidence of M33 by subtype per 100,000 (GUS mid-year population). Dotted line marks the 2010 prevalent-case artefact.

Annual incidence for IIM as a whole and specific subtypes by year is presented in Table 4 and graphically in Figure 3. IIM incidence was lowest in 2020, at 0.875 per 100,000, potentially due to the COVID-19 pandemic, and highest in 2010, at 1.723 per 100,000. The median was 1.209. The median incidence was 0.042 per 100,000 for JDM, 0.303 for DM, 0.561 for PM, and 0.288 for unspecified myopathies. DM, PM, and unspecified myopathies had the lowest incidence in 2020.

This trend was not observed in JDM incidence.

## 4. Discussion

This study analyzed data from the Nationwide General Hospital Morbidity Study (NGHMS), which used the ICD-10 IIM classification. A key strength of this study is its large sample size and nationwide database, with data spanning a long period. Given the rarity of IIMs, a large sample size is vital. This approach allows for a reliable interpretation of incidence, sex distribution, and trends in hospitalization and mortality.

Age and sex distribution characteristics in our cohort are consistent with the available literature [1,17,18]. IIMs, aside from IBM (not included in this study), affect mostly older females (in our cohort, the median age was 56 years for DM and unspecified myopathies and 57 years for PM). Peak incidence in Europe and North America occurs around age 50 [1]. JDM is the exception, as it affects children under 18 years of age [13,14]. Our cohort ranged in age from 6 to 14 years. The overall female-to-male ratio was 1.68 (range 1.44–2.03).

The mean incidence of IIM in the Polish population between 2010 and 2024 was 1.209 per 100,000 per year. This is consistent with similar studies and the available literature [1,2,18]. We observed no marked trend in incidence, except for a dip in 2020 (DM, PM, and unspecified myopathies) and a probable compensatory rise in subsequent years. JDM had the least year-to-year variability.

As is well established in the available literature, PM is a rare and heterogeneous disease. In fact, many cases assigned as PM according to the Bohan and Peter classification are revealed to be other IIMs when serological findings are taken into account [1,2,6]. As a result, only a small number of true PM cases remain. This contrasts sharply with the diagnosis proportions in our cohort, where PM was the most common. The second most frequent diagnosis was other myopathies, reflecting the heterogeneity of IIM patients and the need to implement more detailed classifications, such as the 2017 EULAR criteria, not only in clinical practice but also administratively.

A large study on the Korean population also reported a high prevalence of PM cases. The study included only three IIM diagnoses: PM, DM, and JDM. As the authors themselves admitted, the narrow classification may have contributed to misdiagnosis. The Korean study had a significant advantage, as its database included patient comorbidities, allowing the authors to observe that 88% of IIM patients had more than one comorbidity. This highlights that IIM management is a multifaceted issue. The incidence observed in the Korean population in that study was lower than in our study, at 2.9–5.2 per 1,000,000, and was comparable to studies from Taiwan, Japan, and Israel [17].

Another similar population study in Sweden also reported a relatively high PM prevalence and an IIM incidence of 11 per 1,000,000. This is highly comparable to our results. They differed in their approach by additionally including inclusion body myositis in their classification (coded under G72.4). They also compared incidence across educational backgrounds and found no difference. The authors also found no difference in incidence rates between rural and urban areas, nor a meaningful north-to-south gradient [18].

As the Polish population is predominantly Caucasian, our incidence results may not be comparable to those of other ethnicities. Ethnic background and differences may explain variability in incidence across countries.

Hospitalization rates for all subtypes remained stable over the time period, with a small dip in 2020. On the other hand, mortality rates rose for DM, unspecified myopathies, and PM in the year 2020. This was possibly due to the COVID-19 pandemic. Patients with IIM, especially those with ILD, are at higher risk of developing COVID-19 and its severe form [19,20]. Despite this, overall outcomes in such patients are not worse [19]. Perhaps the rise in mortality rates can be attributed to poorer access to healthcare during the pandemic, resulting in delayed diagnosis and treatment. The available data do not allow for definitive conclusions.

The incidence of specific IIM subtypes across voivodeships was similar. The highest incidence of JDM, PM, and unspecified myopathies was observed in the Podkarpackie Voivodeship, and the highest incidence of DM was observed in the Opolskie Voivodeship. It is difficult to draw meaningful conclusions because the differences in incidence rates are not stark.

A limitation of this study is the classification, which recognizes only DM, PM, JDM, and “unspecified myopathies”. This naturally leads to the underdiagnosis and misdiagnosis of IIM subtypes so that they fit the narrow criteria. In particular, overlap myositis and other currently recognized IIMs, as distinguished in the newer classifications, are coded as DM, PM, JDM, or unspecified, depending entirely on the case characteristics and the doctor’s judgment. This obscures the true diagnosis types and overrepresents others, especially PM, which for many years was used as an umbrella term for what are now recognized IIM subtypes [1,2,6]. Limited access to more detailed medical histories (particularly serological markers) and the very large population studied also prevented us from rediagnosing patients according to modern classifications. This affects our findings, particularly the numerical data. However, other large nationwide studies similar to ours [17,18] have also used the ICD-10 classification, as it is internationally recognized and officially implemented by WHO member countries. ICD-10 is the standard for public health statistical purposes. The International Statistical Classification of Diseases and Related Health Problems, 11th Revision (ICD-11), was only created in 2022 and is planned to be implemented in Poland before 2027. As such, data gathered between 2010 and 2024 could not be coded using the newer revision or the detailed 2017 EULAR and/or Gianni et al. classifications.

Another factor that may contribute to discrepancies in the number of diagnoses is that IIMs are rare diseases, and the original, well-known Bohan and Peter classification of 1975 [8] is often used in everyday clinical practice when clinicians code diagnoses in electronic documentation. This may also result in the overrepresentation of DM and PM.

Another limitation of this study is its retrospective design and reliance on the NGHMS. We were very careful in selecting and analyzing data to exclude any cases suspected of coding inaccuracies. The database lacked much valuable clinical information that could have enriched the conclusions drawn. In particular, the absence of MSA types and their occurrence prevents us from assessing their influence on hospitalization and mortality rates. Because they are associated with specific clinical features, malignancy, mortality, and organ involvement, including MSA types could provide a more detailed and, importantly, different view of the data. This remains an area for future research.

Similarly, data on the occurrence of malignant comorbidities, organ involvement, or interventions performed could not be gathered. This further impacts data gathered on patient mortality, as there is no way to distinguish the particular reasons for patients’ passing—whether they were due to a co-occurring malignancy, heavy pulmonary involvement, or different aspects of IIM clinical manifestations. As such, our observations and conclusions on mortality rates are preliminary and may change.

## 5. Conclusions

In conclusion, we observed stable incidence and hospitalization rates of IIM in Poland between 2010 and 2024, both overall and across its four subtypes. Nationwide, comprehensive data are an extremely valuable tool for researching and understanding diseases as rare as IIMs.

## Figures and Tables

**Table 1:** Shows the patient cohort characteristics.

Characteristic	n	%
Patients in cohort	7139	100
Female	4475	62.7
Male	2664	37.3
Age < 18 years	776	10.9
Age ≥ 18 years	6362	89.1

Female-to-male ratio: 1.68:1 (binomial *p* < 10^−100^). Median age: 55 years (IQR 39–66).

**Table 2:** Shows detailed patient cohort characteristics, including sex and age, in relation to diagnosis type.

ICD-10 Subtype	n	% of Cohort	Female n (%)	Male n (%)	F:M	Age, years Median (IQR)	Age, years Mean ± SD	<18/≥18 years n	LOS, d Median (IQR)
M33.0 Juvenile dermatomyositis	270	3.8	181 (67.0)	89 (33.0)	2.03	11 (6–14)	10.3 ± 4.4	270/0	5 (2–10)
M33.1 Other dermatomyositis	1919	26.9	1276 (66.5)	643 (33.5)	1.98	56 (41–66)	52.5 ± 19.1	128/1791	8 (4–13)
M33.2 Polymyositis	3220	45.1	1903 (59.1)	1317 (40.9)	1.44	57 (41–67)	52.4 ± 20.0	280/2939	8 (4–12)
M33.9 Dermatopolymyositis, unspecified	1730	24.2	1115 (64.5)	615 (35.5)	1.81	56 (41–66)	52.5 ± 18.5	98/1632	7 (4–12)
All M33	7139	100	4475 (62.7)	2664 (37.3)	1.68	55 (39–66)	50.9 ± 20.6	776/6362	7 (4–12)

Sex × subtype: χ^2^ = 34.1, df = 3, *p* = 1.9 × 10^−7^ (Cramér’s V = 0.069); female predominance is the weakest in polymyositis (M33.2). Age × subtype: Kruskal–Wallis *p* = 1.1 × 10^−141^; Dunn test: M33.0 was younger than all others (*p* < 0.001); the three adult forms do not differ.

**Table 3:** Shows hospitalization characteristics, duration, mortality and sex ratio in relation to diagnosis type.

ICD-10 Subtype	LOS, d Median (IQR)	Deaths n (%)	Deaths F/M n	Age at Death Median (IQR)
M33.0: Juvenile dermatomyositis	5 (2–10)	0 (0.00)	0/0	—
M33.1: Other dermatomyositis	8 (4–13)	35 (1.82)	19/16	63 (51–74)
M33.2: Polymyositis	8 (4–12)	51 (1.58)	30/21	69 (63–80)
M33.9: Dermatopolymyositis, unspecified	7 (4–12)	32 (1.85)	21/11	73 (59–79)
All M33	7 (4–12)	118 (1.65)	70/48	68 (58–78)

LOS × subtype: *p* = 1.6 × 10^−10^. Median LOS: 7 d (IQR 4–12). In-hospital mortality: 118/7139 (1.65%). Multivariable logistic regression (death ~ age + sex): age OR: 1.063 per year (95% CI 1.048–1.078, *p* = 1.6 × 10^−17^); female OR: 0.695 (95% CI 0.478–1.012, *p* = 0.058). Subtype omitted from the model because M33.0 had zero deaths (perfect separation). LOS = length of stay.

**Table 4:** Shows the median annual incidence per year and by diagnosis type.

Year	All M33	M33.0 Juvenile DM	M33.1 Other DM	M33.2 Polymyositis	M33.9 Unspecified
2010	1.723	0.134	0.566	0.683	0.340
2011	1.313	0.055	0.426	0.504	0.330
2012	1.170	0.034	0.376	0.488	0.272
2013	1.288	0.034	0.312	0.634	0.309
2014	1.185	0.023	0.385	0.507	0.270
2015	1.241	0.052	0.299	0.622	0.268
2016	1.312	0.044	0.289	0.653	0.325
2017	1.207	0.036	0.258	0.554	0.359
2018	1.211	0.042	0.307	0.568	0.294
2019	1.123	0.031	0.297	0.513	0.281
2020	0.875	0.044	0.217	0.379	0.235
2021	0.995	0.042	0.261	0.422	0.269
2022	1.120	0.050	0.249	0.572	0.249
2023	1.355	0.040	0.369	0.603	0.343
2024	1.563	0.045	0.411	0.726	0.381
Median (2011–2024)	1.209	0.042	0.303	0.561	0.288

Incidence = new first hospitalizations/GUS mid-year population. A dip in 2020 (COVID-19) recovered above pre-pandemic levels by 2024.

## Data Availability

No new data were created or analyzed in this study. Data sharing is not applicable to this article.

## Abbreviations

IIM	idiopathic inflammatory myopathies
DM	dermatomyositis
CADM	clinically amyopathic dermatomyositis
PM	polymyositis
JDM	juvenile dermatomyositis
AsyS	antisynthetase syndrome
OM	overlap myositis
IMNM	immune-mediated necrotizing myositis
IBM	inclusion body myositis
NGHMS	Nationwide General Hospital Morbidity Study
ILD	interstitial lung disease
MAA	myositis-associated antibodies
MSA	myositis-specific antibodies
anti-Jo-1	anti-histidyl tRNA synthetase antibody
anti-Mi-2	anti-Mi-2 antibody
anti-MDA5	anti-melanoma differentiation-associated gene 5 antibody
anti-NXP2	anti-nuclear matrix protein 2 antibody
anti-TIF1	anti-transcription intermediary factor 1 antibody
PLCL1	phospholipase C-like 1
BLK	B lymphoid tyrosine kinase
